# euPrevent

**DOI:** 10.1093/eurpub/ckac129.487

**Published:** 2022-10-25

**Authors:** B Van Der Zanden

**Affiliations:** euPrevent, Heerlen, Netherlands

## Abstract

euPrevent is a non-profit organisation and a Euroregional network that wants to promote the quality of life of citizens in the border regions of the Netherlands, Germany and Belgium. Policy in the field of care and well-being is mainly organised at national level. This has a major impact on citizens in the border region who regularly cross the border for work or family reasons. We therefore focus on the establishment of sustainable, cross-border network connections between health organisations in the border region. Our cross-border cooperation is based on the following three frameworks, within we work: (1) Positive Health, (2) Health in All Policies (HiAP), and (3) Sustainable Development Goals. The network will be represented by Brigitte van der Zanden, the Director of euPrevent and a long standing expert in cross-border health issues.

